# Bolometric IR photoresponse based on a 3D micro-nano integrated CNT architecture

**DOI:** 10.3762/bjnano.15.84

**Published:** 2024-08-15

**Authors:** Yasameen Al-Mafrachi, Sandeep Yadav, Sascha Preu, Jörg J Schneider, Oktay Yilmazoglu

**Affiliations:** 1 Department of Electrical Engineering and Information Technology, Institute for Microwave Engineering and Photonics (IMP), Technical University of Darmstadt, 64283 Darmstadt, Germany; 2 Eduard-Zintl-Institut für Anorganische und Physikalische Chemie, Technical University of Darmstadt, 64287 Darmstadt, Germany

**Keywords:** 3D micro-nano architecture, blackbody absorption, CNT microbolometer, high responsivity, high spatial resolution

## Abstract

A new 3D micro-nano integrated M-shaped carbon nanotube (CNT) architecture was designed and fabricated. It is based on vertically aligned carbon nanotube arrays composed of low-density, mainly double-walled CNTs with simple lateral external contacts to the surroundings. Standard optical lithography techniques were used to locally tailor the width of the vertical block structure. The complete sensor system, based on a broadband blackbody absorber region and a high-resistance thermistor region, can be fabricated in a single chemical vapor deposition process step. The thermistor resistance is mainly determined by the high junction resistances of the adjacent aligned CNTs. This configuration also provides low lateral thermal conductivity and a high temperature coefficient of resistance (TCR). These properties are advantageous for new bolometric sensors with high voltage responsivity and broadband absorption from the infrared (IR) to the terahertz spectrum. Preliminary performance evaluations have shown current and voltage responsivities of 2 mA/W and 30 V/W, respectively, in response to IR (980 nm) absorption for a 20 × 20 μm^2^ device. The device exhibits an exceptionally fast response time of ≈0.15 ms, coupled with a TCR of −0.91 %/K. These attributes underscore its high operating speed and responsivity, respectively. In particular, the device maintains excellent thermal stability and reliable operation at elevated temperatures in excess of 200 °C, extending its potential utility in challenging environmental conditions. This design allows for further device miniaturization using optical lithography techniques. Its unique properties for mass production through large-scale integration techniques make it important for real-time broadband imaging systems.

## Introduction

Non-cryogenic infrared (IR) microbolometers represent a cutting-edge technology with numerous advantages at room temperature operation. New thermally isolated micro-nano architectures equipped with integrated temperature sensors should outperform their predecessors regarding various critical aspects, such as ultra-broadband detection from ultraviolet (UV) to terahertz radiation [1], increased responsivity, unwavering reliability, ultrafast response time, and a substantial reduction in device size. The introduction of large, two-dimensional (2D) detector arrays has further increased the complexity of the detector optimization. In response to these multifaceted challenges and the quest for non-cryogenic IR microbolometer technology, carbon nanotubes (CNTs) have emerged as highly promising candidates [2] with broadband blackbody absorption [3], high resistance perpendicular to the CNT orientation [4], low lateral thermal conductivity [5], a high temperature coefficient of resistance (TCR) [6], and high mechanical stability [7].

Vertically aligned CNTs (VACNTs) are considered to be nearly ideal blackbody absorbers because of their ability to absorb light over a broad spectral range (0.2–200 μm), closely resembling the theoretical blackbody that absorbs all wavelengths of light [3,8–10]. This performance is based on the high absorption of VACNTs coupled with their low reflectance over this broad spectrum. Vertical alignment and distribution of the CNTs are critical to achieving such efficient absorption, making VACNTs effective blackbody analogs for broad-spectrum applications [3]. The device resistance perpendicular to the CNT orientation is mainly determined by the high junction resistances of the neighboring aligned CNTs [4]. This configuration allows for high device resistance, low thermal conductivity, and high temperature coefficient of resistance, thus, enabling customized specific requirements for various applications. While the thermal conductivity along the CNT orientation is large, it decreases dramatically in the perpendicular direction [5]. This thermal behavior varies significantly depending on structure, density, and fabrication of the CNTs. Single-walled CNTs (SWCNTs) have achieved thermal conductivities as high as 6600 W/mK in the CNT orientation, in sharp contrast to multiwalled CNT (MWCNT) bundles, which can have thermal conductivities as low as less than 0.1 W/mK perpendicular to the CNT orientation, making them viable as thermal insulators.

At the core of non-cryogenic IR microbolometers is the fundamental principle of absorbing incident IR radiation and converting it to heat. This conversion process results in a change in the resistance of the active material, which, in turn, is related to the temperature coefficient of resistance (TCR) of the material. The electrical conductivity of a vertically aligned CNT structure is defined by the intrinsic conductivity along the CNTs and the tunneling at the CNT junctions. The vertical electrical conduction is based on long conduction paths along the CNTs and few junction contacts, while the lateral electrical conduction is based on short conduction paths along the CNTs (length between junction contacts) and many junction contacts. Experimental studies have shown that the TCR is negative for aligned CNTs [6]. The main factor influencing the TCR of a network is the TCR of the CNTs themselves. In particular, larger negative TCRs can be achieved if the conduction along the CNTs in the effective conduction path remains small, as is the case for lateral electrical conduction paths [6]. VACNTs with mainly lateral electrical conduction paths can satisfy this condition. These properties make CNTs highly attractive options for non-cryogenic IR microbolometer and thermal detection technology.

The sensitivity of a bolometer is quantified by its responsivity, which is given by the expression [11]:


[1] $$R_{V}=\frac{\Delta V}{\Delta P}=\frac{I_{\text{bias}}R\alpha \eta }{G\sqrt{1+\omega^{2}\tau^{2}}},$$

where Δ*V* is the corresponding voltage change, Δ*P* represents the incident power on the active detection area, *I*_bias_ is the bias current, *R* is the resistance of the bolometer, α = (d*R*/d*T*)/*R* is the TCR, η is the absorption efficiency, *G* is the thermal conductance to the substrate, ω is the angular modulation frequency, τ = 1/(2π*f*_cut−off_) is the time constant of the detector, and *f*_cut−off_ is the frequency at 70% responsivity. Based on this equation, efficient bolometers require high optical absorption, a high TCR, good thermal isolation from the environment (characterized by a small value of *G*), and a small time constant.

The research presented in this paper provides a 3D CNT design with micro-nano integrated lateral contacts. The lateral and vertical dimensions of the M-shaped CNT architecture can be adjusted independently to achieve low thermal conductivity, high lateral resistance, and small response time to achieve a higher bolometric effect. Our first responsivity measurements with similar CNT wall structures were presented in the conference proceedings [12]. This configuration 1 (called sample 1) had additional parasitic contact resistances to the M-shaped CNT block. The new configuration 2 (called sample 2) used direct Au whisker contacts to the CNT block with much lower contact resistances for reliable measurements. The true performance of the original M-shaped CNT block with higher TCR, higher voltage, and higher current responsivity could be demonstrated. In this work, new noise measurements were performed and compared for both configurations. The new configuration 2 is superior regarding the optimization of the M-shaped bolometer design. The final design with a micro-nano integration using optimized contacts between the Cr/Au pads and the M-shaped CNT block can then be achieved with additional CNT blocks on the Cr/Au pads as shown in [7].

Furthermore, this research embarks on a comprehensive exploration of the potential for large-scale production of CNT-based non-cryogenic IR microbolometers and thermal detectors, promising cost-effective solutions ready for widespread adoption across multiple industries. It represents a significant step forward in addressing the growing demand for advanced thermal sensing solutions and sets the stage for transformative advances in non-cryogenic IR microbolometer and thermal detector technology. The following sections provide a comprehensive overview of the 3D micro-nano design, fabrication methods, performance characteristics, and potential applications. Simultaneously, emphasis will be placed on the effective conversion of heat into electrical signal, which is the most critical effect contributing to the overall success of non-cryogenic IR microbolometer and thermal detector design [11].

## Experimental

**Figure 1:**
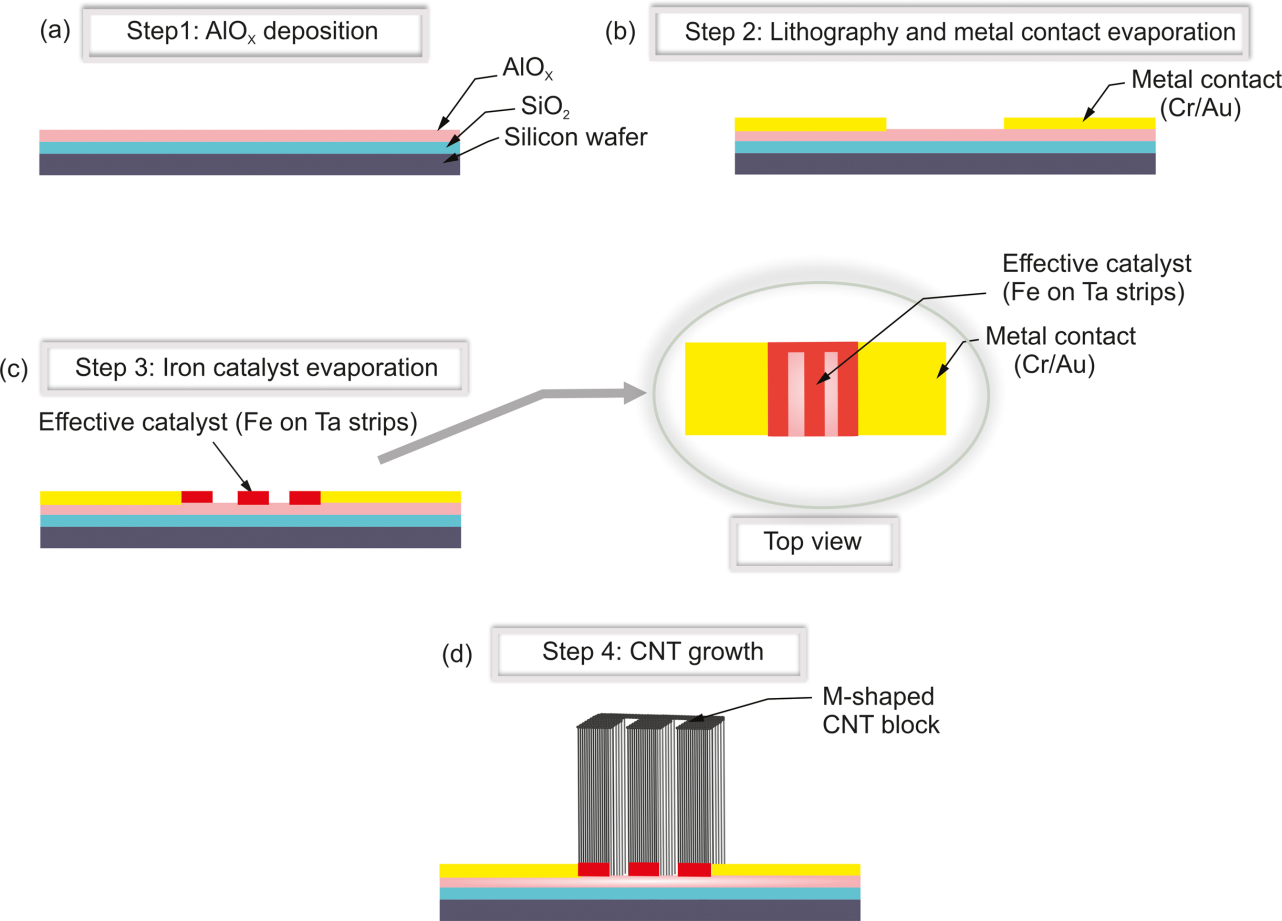
Fabrication process of the pixel-based CNT microbolometer. (a) AlO*_x_* deposition, (b) lithography process and metal contact evaporation, (c) iron catalyst evaporation, and (d) CNT growth.

**Figure 2:**
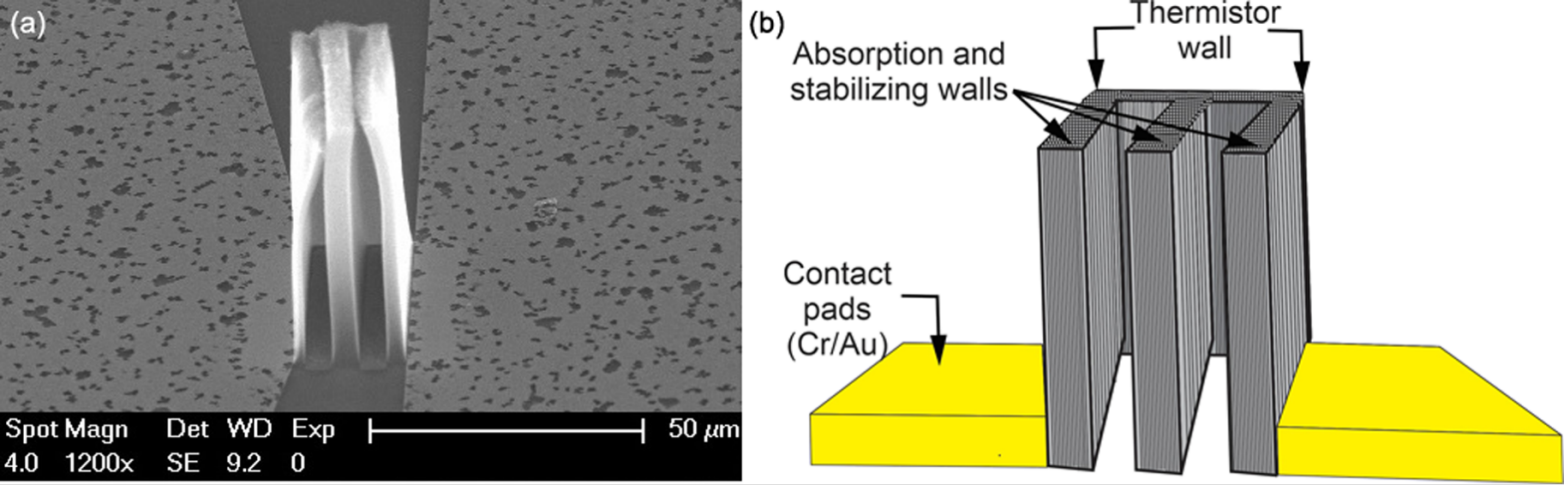
(a) Scanning electron microscopy image and (b) 3D close-up of the pixel-based CNT microbolometer.

The process started with cleaning a silicon substrate with a 600 nm thick thermally oxidized SiO_2_ layer. The wafer was p-type and lightly boron-doped (Si-Mat, Silicon Materials). The substrate was thoroughly cleaned to remove impurities and contaminants to provide an ideal condition for CNT growth. Subsequently, an essential 30 nm aluminum oxide (AlO*_x_*) layer was deposited by atomic layer deposition to support the elongated growth of CNTs (Figure 1a). The contact pad regions were opened by an optical lithography process prior to the evaporation of Cr/Au (20 nm, e-beam/100 nm, thermal) (Figure 1b). The overall M-shape for the CNT growth as shown in Figure 1c was defined by overlapping two tantalum-based growth stop strips (150 nm, sputtering) with a rectangular Fe layer (2 nm, e-beam) on the Si wafer. Only regions with direct Fe/AlO*_x_* contact were active for CNT growth. This combination allows for the definition of any shape, offering versatility for optimizing device parameters such as resistance, optical absorption characteristics, and heat conductivity. The M-shape was used to have a homogeneous gas flow parallel to the CNT walls during CNT growth to achieve repeatably aligned walls. Prior to CNT growth, the Fe catalyst layer self-assembled into Fe nanoparticles at ≈750 °C. Finally, the samples were synthesized by water-assisted chemical vapor deposition (CVD) at 800 °C, similar to the CVD process presented in [13–14], to achieve a crystalline graphitic nature of the carbon nanotubes. Argon was used as the carrier gas and ethylene as the carbon source. A stream of water vapor acted as a catalyst activator. The height of the CNT bundles (30–60 μm) depended on the CVD growth time. CNT bundles with a height of ≈40 μm were grown in ca. 1 min as shown in Figure 2a.

The resulting M-shaped VACNTs have an initial pixel dimension of 20 × 20 μm^2^ with a ≈1 μm thick CNT thermistor wall stabilized by complementary CNT block structures. Two strategically positioned metal electrodes on the Si substrate provide lateral contacts with the vertical CNT bolometer, facilitating effective resistance measurements. Importantly, this CNT structure exhibits remarkable thermal stability, retaining reliability at temperatures reaching up to 200 °C. This exceptional thermal resilience makes it ideally suited for demanding high-temperature applications, showing its potential across various industries.

**Figure 3:**
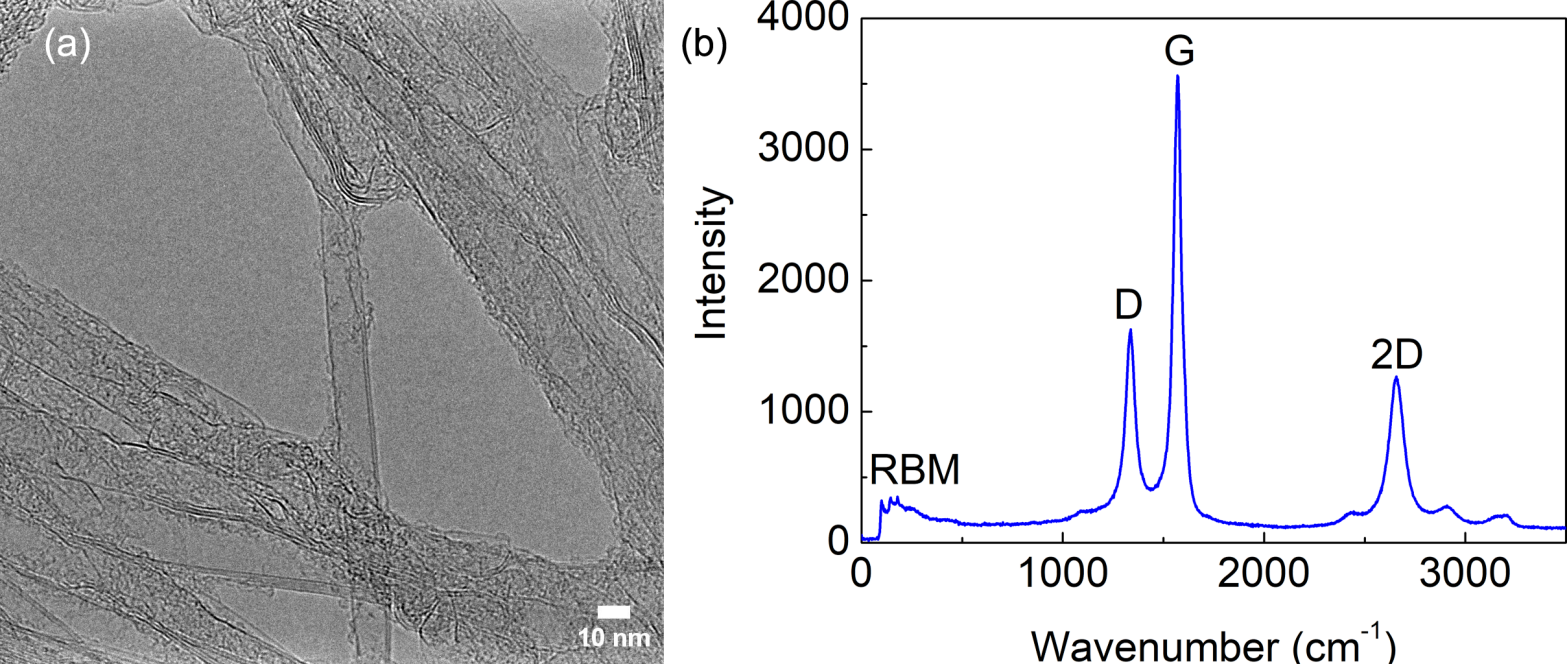
(a) TEM image showing DWCNTs and (b) Raman spectrum of the VACNTs.

Transmission electron microscopy (TEM) and Raman characterizations of the VACNTs from similar CNT growth conditions have been published in previous works [7,15]. It was shown that more than 90% of the CNTs grown on the Si wafer were double-walled carbon nanotubes (DWCNTs). The new TEM micrograph in Figure 3a shows the DWCNTs. Diameter and chirality of the carbon nanotubes are strongly influenced by the size and morphology of the catalyst particles. A correlation between cluster size and diameter of the CNT grown on it was shown in [15]. The effect of the growth temperature on the diameter distribution and chirality of single-walled carbon nanotubes can be found in [16]. A new Raman spectrum was recorded in the range of 50 to 3500 cm^−1^ using an excitation wavelength of 488 nm (see Figure 3b). It shows the main modes (G, D, and 2D) typical of all carbon nanotubes and a less intense radial breathing mode (RBM). The G-band peak corresponds to the crystalline graphitic nature of the carbon nanotubes, while the D- and 2D-band peaks are the defect band peak and its first overtone, respectively. At lower wavenumbers, RBM peaks are seen. Their appearance symbolizes the presence of few-layer CNTs (single or double layer).

Compared to SWCNTs [8], for which the characteristic absorption peaks depend on the nanotube diameter, DWCNTs have a relatively flat spectral absorption due to the excitonic transition energies of the inner and outer tubes [9]. The broadband absorption of our fabricated block structures has not yet been measured. However, the absorption of similarly grown VACNTs (water-assisted CVD on silicon substrates with ethylene as the carbon source) has been investigated by [3]. They showed that vertically aligned SWCNTs can absorb light almost perfectly with a reflectance of 0.01–0.02 over a very wide spectral range (0.2–200 μm). The UV-to-mid-IR absorption characteristics showed little variation with CNT heights ranging from 2 to 460 μm [3]. The authors attributed this blackbody behavior to the sparseness and imperfect alignment of the vertical SWCNTs, which suppressed reflection. Due to multi-reflection in the CNT forest and broadband absorption in the single CNTs [8], the effective reflection from the forest decreased very rapidly. MWCNTs have also been used as absorbers in the infrared region (2–20 μm) with an optical absorption coefficient above 90% [10]. Similar values were achieved for different synthesis temperatures (500–700 °C) with different CNT diameters, qualities, and heights. So far, we have only investigated the IR response. Far-IR and THz measurements will be carried out in the future to prove the broadband property of our new bolometer architecture.

## Results

**Figure 4:**
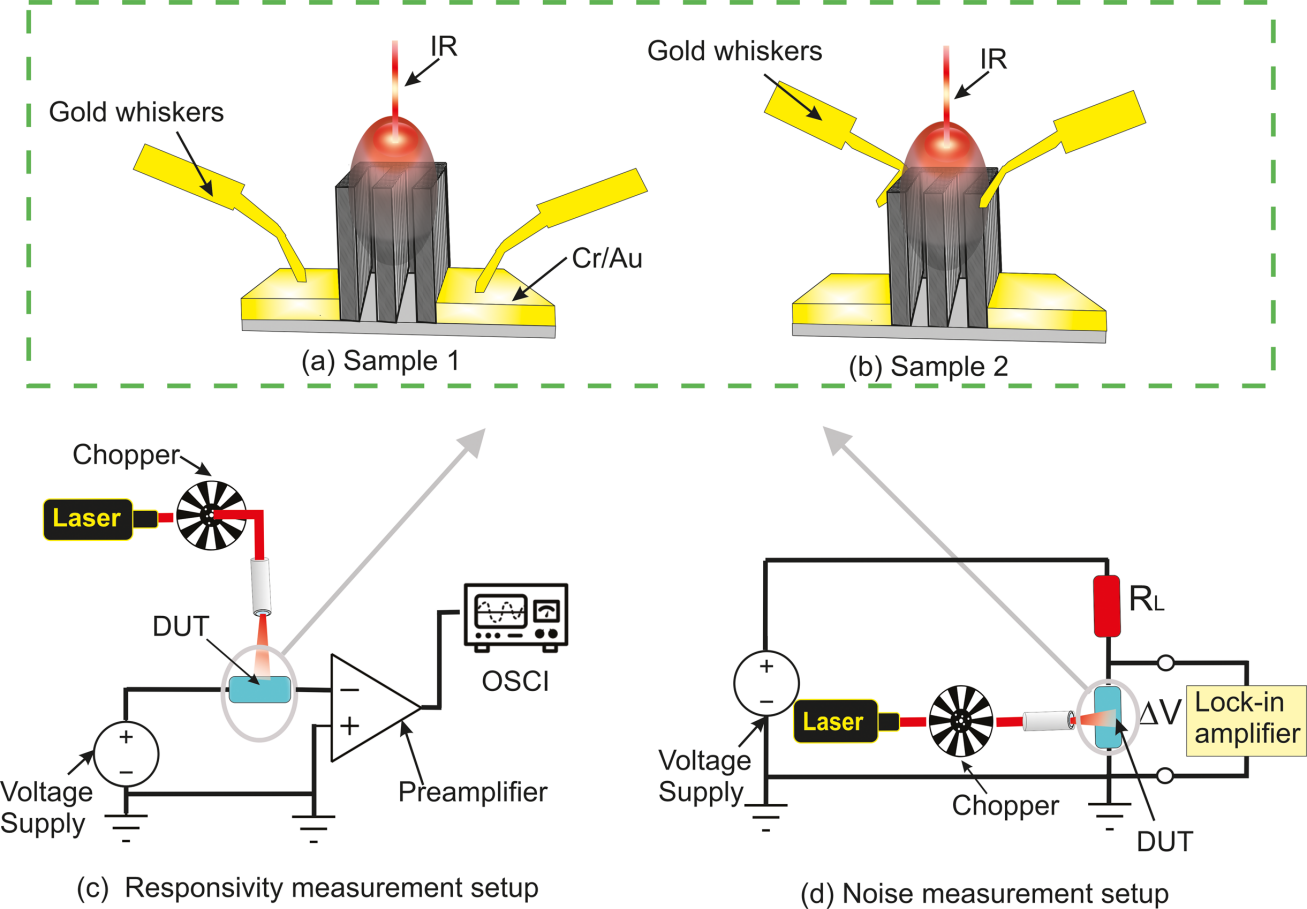
Schematic of the device under test (DUT) and measurement setups to characterize the CNT-based microbolometer. (a) Sample 1 with metal contact pads and (b) sample 2 with direct contacts using gold whiskers with a diameter of 20 μm. (c) Responsivity measurement setup using a preamplifier and (d) responsivity as well as noise measurement setup using a lock-in amplifier. Panels (a) and (b) do not represent the real dimensional relationships.

The experimental characterization evaluated a number of parameters, including temperature coefficient of resistance (TCR), effective thermal conductance (*G*_th_), current responsivity ℜ*_I_* = Δ*I*/*P*_in_, which is the output current change per incident power, voltage responsivity ℜ*_V_* = Δ*V*/*P*_in_, which is the output voltage change per incident power, and noise equivalent power (NEP), which is the minimum power that can be detected by the bolometer for a 1 Hz detection bandwidth. The experimental setups for the responsivity and noise measurements included several devices, such as a DC power supply, an IR laser source, an optical chopper (Thorlabs MC2000B), a low-noise transimpedance amplifier (TIA), model HMS 564, an oscilloscope (Tektronix TDS), and a lock-in amplifier (Stanford Research Systems SR510), as shown in Figure 4c,d. The oscilloscope data was read by a computer.

The responsivity of the CNT-based microbolometer was characterized using a continuous-wave laser source emitting radiation at 980 nm. A Thorlabs compact laser diode controller set the output power to 4 mW. The spot size of the laser beam of 100 μm was accurately determined using the knife edge method [17]. A mechanical chopper was used to modulate the laser beam intensity. The key component in the responsivity measurement setup, as shown in Figure 4c, is a low-noise TIA with a gain of 10^5^ V/A [12]. Its output was connected to the oscilloscope. The responsivity was measured at chopper frequencies between 90 and 1100 Hz. To determine the noise floor, we redesigned the post-detection electronics by replacing the low-noise TIA and oscilloscope with a lock-in amplifier (Stanford-510), as shown in Figure 4d. The lock-in amplifier was synchronized to the optical chopper frequency.

The fabricated M-shaped samples shown in Figure 4 were characterized either by the integrated metal contact pads (configuration 1, called sample 1) or by the use of gold whiskers for direct electrical contact (configuration 2, called sample 2). Sample 1 in a shows a bolometer configuration with lateral contact pads for micro-nano integration with potential for high integration densities. However, the initial devices in this configuration 1 had an additional parasitic contact resistance to the M-shaped CNT block. Sample 2 in b with whisker contacts was used to reduce the parasitic series resistance, increase the responsivity, and reduce the noise. Therefore, configuration 2 showed the true performance of the original M-shaped CNT block. Optimized contacts between the Cr/Au pads and the M-shaped CNT block can be obtained, for example, with additional CNT blocks grown on the Cr/Au pads, as shown in [7].

### DC measurements

**Figure 5:**
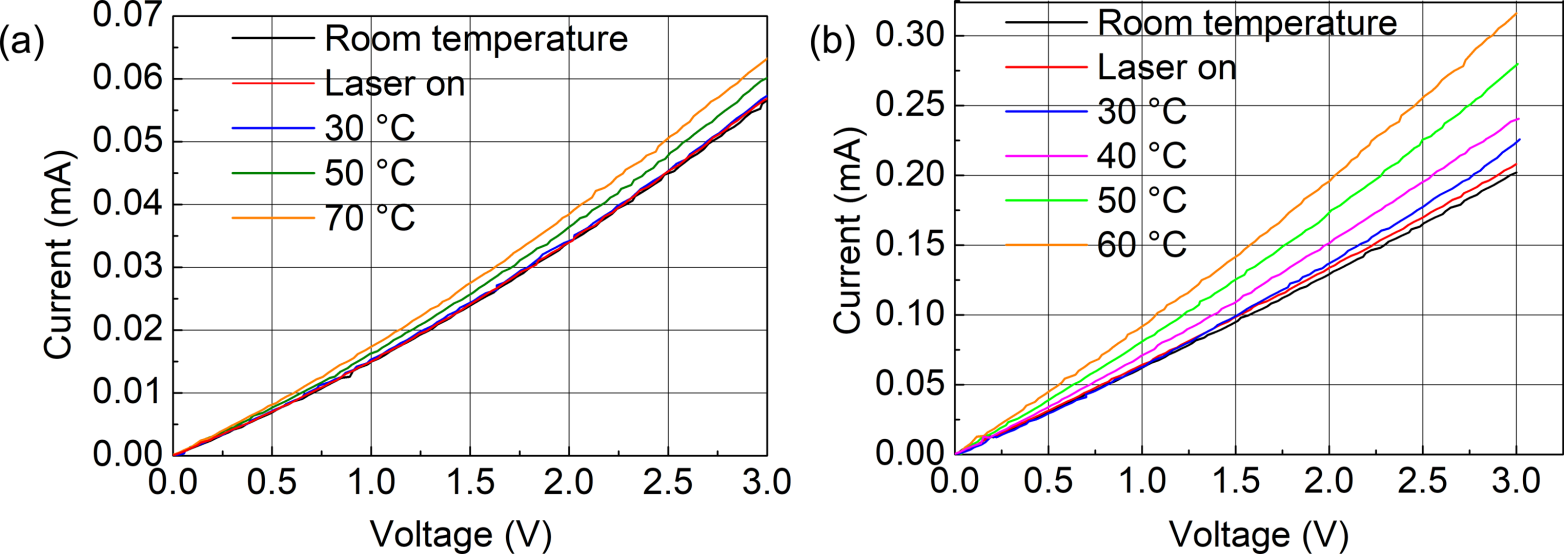
Temperature-dependent *I*–*V* curves (a) for sample 1 with contact pads and (b) for sample 2 using gold whisker contacts.

**Figure 6:**
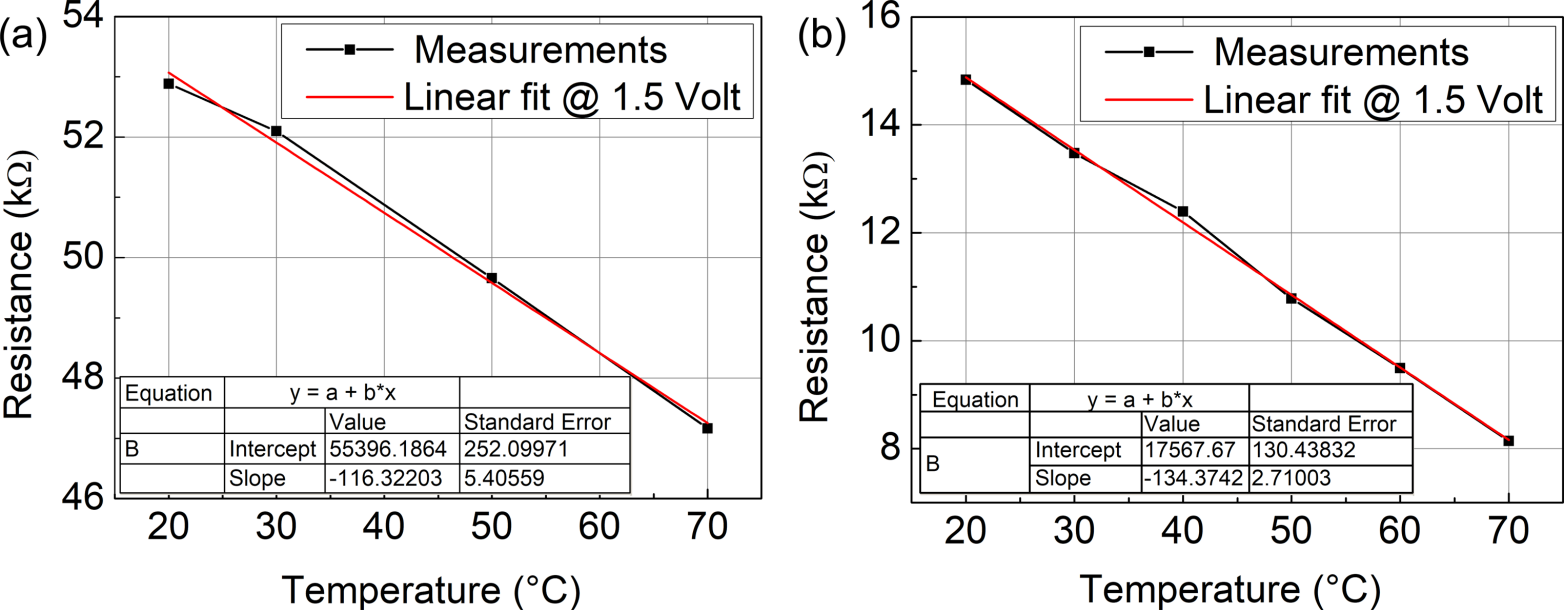
Resistance–temperature relationship (a) for sample 1 with contact pads and (b) for sample 2 using gold whisker contacts.

The first step involved the measurement of the bolometer resistance *R*_bolometer_ as a function of temperature *T* induced by a hot plate as well as the modulated laser beam [18]. Both configurations, with metal contact pads (sample 1) and direct gold whisker contacts (sample 2), were characterized. The resulting *I*–*V* curves and the device resistance as a function of the temperature are shown in Figure 5 and Figure 6, respectively. The room-temperature resistances *R*_01_ (sample 1) and *R*_02_ (sample 2) at a bias voltage *V*_bias_ = 1.5 V were 53.1 and 14.8 kΩ, respectively. The linear temperature dependence is given by the following equation [18–19]:


[2] $$R\left(T\right)=R_{0}\left[1+\alpha \left(T-T_{0}\right)\right],$$

where *R*(*T*) is the resistance at temperature *T*, *R*_0_ is the resistance at room temperature *T*_0_ = 20 °C, α is the TCR, and *T* is the temperature. The slope of *R*(*T*) in Figure 6 is Δ*R*/Δ*T* = *R*_0_·TCR. The TCR can be calculated as:


[3] $$\text{TCR}=\frac{\Delta R}{R_{0}\Delta T}.$$

This equation quantifies how a device’s resistance changes with temperature, providing valuable information about temperature sensitivity. The TCRs for sample 1 and sample 2 at 1.5 V were −0.22 and −0.91 %/K, respectively. This confirms the experimental studies with negative TCR for aligned CNTs [4,6]. A larger negative TCR was achieved for sample 2 with reduced series resistance to the M-shaped CNT block. For the calculation of the thermal conductance *G*_th_ the temperature increase due to the electrical power dissipation in the bolometer *P*_in_ was used. The temperature increase Δ*T* was calculated from the resistance change shown in Figure 5. The equation Δ*T* = *P*_in_/*G*_th_ can be rewritten and used for the calculation of the thermal conductance *G*_th_:


[4] $$G_{\text{th}}=\frac{V_{\text{bias}}^{2}}{R_{\text{bolometer}}\left(V_{\text{bias}}\right)\Delta T},$$

where *V*_bias_ is the bias voltage of 1.5 V, and *R*_bolometer_(*V*_bias_) is the resistance at bias voltage. The temperature change Δ*T* due to the electrical power dissipation was calculated using Equation 2, and the resistance change in Figure 5 for sample 1 and sample 2 was determined using temperature values of 129.1 and 20.3 °C, respectively. The thermal conductances of sample 1 and sample 2 were calculated as *G*_th1_ = 7.45 × 10^−6^ W/K and *G*_th2_ = 3.3 × 10^−7^ W/K, respectively.

### Responsivity measurement

**Figure 7:**
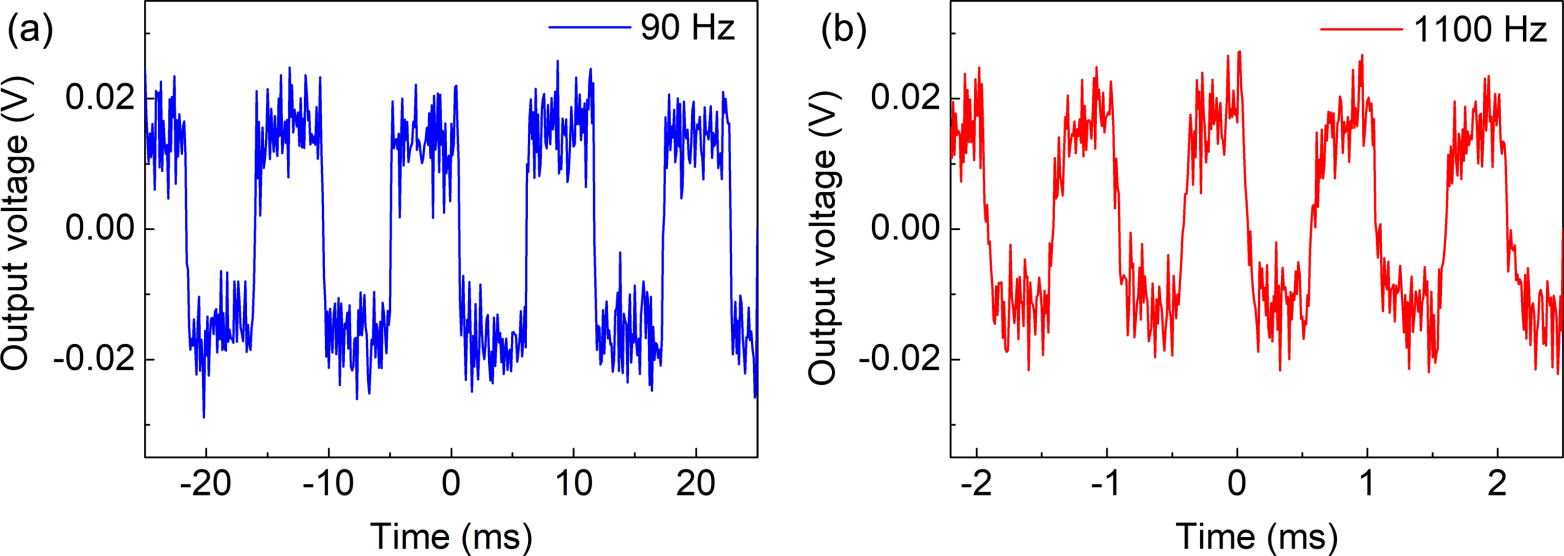
Response measurements for sample 1 using metal contact pads at (a) 90 Hz and (b) 1100 Hz (with an average of 10 measurements). The standard deviation of the peak σ is 18 mV.

**Figure 8:**
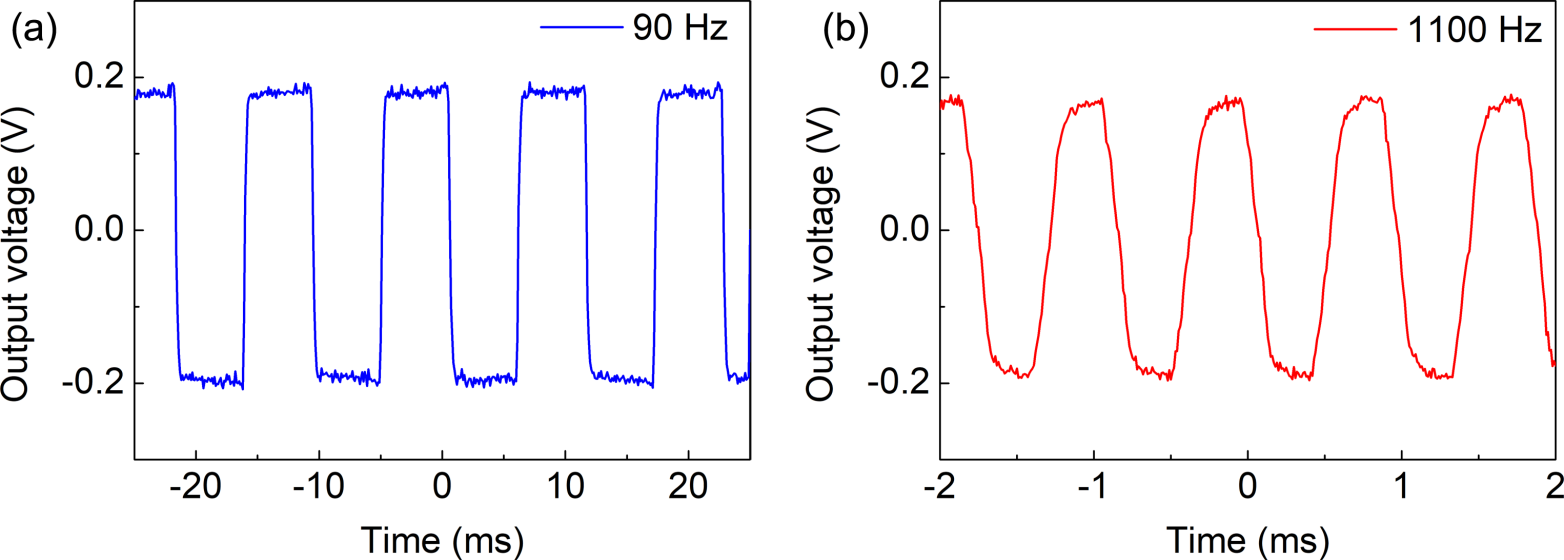
Response measurements for sample 2 using gold whisker contacts at (a) 90 Hz and (b) 1100 Hz (with an average of 10 measurements). The standard deviation of the peak σ is 22 mV.

The voltage responsivity (ℜ*_V_*) is one of the main device parameters in the evaluation of microbolometer performance, serving as a quantitative measure of the device’s ability to transduce absorbed IR radiation into an electrical signal [18]. Regarding CNT-based microbolometer systems, the responsivity is influenced by a number of factors inherent to the CNT material and the device architecture. Both samples were characterized using the readout circuit comprising the TIA and an oscilloscope to record their responses. The output voltage change Δ*V* at the TIA was measured at modulating frequencies of 90 and 1100 Hz as shown in Figure 7 and Figure 8. Sample 1 and sample 2 have shown voltage changes of ≈40 and ≈320 mV, respectively. Other devices measured at additional frequencies are described in Supporting Information File 1, Figures S3–S5.

The laser power density *P*/*A* = 4 × 10^6^ W/m^2^ was almost uniform on the device area of *A* = 20 × 20 μm^2^. The total incident power *P*_in_ = 1.6 mW was calculated by multiplying the power density *P*/*A* with the device area *A*. The current change Δ*I* induced by this power was calculated from the voltage change Δ*V* in Figure 7 and Figure 8 using the system’s amplification factor Δ*I* = (Δ*V*)/(10^5^ V/A). The temperature increases under IR illumination were Δ*T* = 1.3 °C and Δ*T* = 2 °C for sample 1 and sample 2, respectively, as shown in Figure 5. Current responsivity ℜ*_I_* = (Δ*I*)/(*P*_in_) and voltage responsivity ℜ*_V_* = ℜ*_I_*·*R*_bolometer_ calculated for sample 1 and sample 2 were 0.25 mA/W (13 V/W) and 2 mA/W (30 V/W), respectively. Using the oscilloscope data at 1100 Hz chopper frequency, the response time (defined as the time taken for the signal amplitude to rise from 10% to 90% of the maximum value) was found to be a ≈0.15 ms for both samples.

### Noise measurement

In addition to the calculations of TCR, *G*_th_, and responsivity, noise voltage *V*_n_ measurements were performed to evaluate the noise characteristics of the new CNT microbolometer for both configurations. A lock-in amplifier was used for the noise *V*_n_ measurements as shown in Figure 4d. The NEP value was calculated using the following equation [18]:


[5] $$\text{NEP}=\frac{V_{\text{n}}}{{ℜ}_{V}},$$

where ℜ*_V_* is the voltage responsivity of the microbolometer. The noise voltages of sample 1 and sample 2 measured at 90 Hz were 40.6 and 16.6 μV, respectively. They were almost constant at chopper frequencies ≥60 Hz. The calculated NEP values for sample 1 and sample 2 were 3.12 and 0.55 μW·Hz^−0.5^, respectively. The decreased contact resistance in sample 2 with direct Au whisker contacts resulted in lower noise values, indicating the importance of the final contact resistance.

## Discussion

Several CNT architectures (pristine or in composites) have shown photoelectric or bolometric effects [20–21]. Our new bolometers with both configurations were compared to state-of-the-art bolometer architectures with aligned and pristine CNTs, which were used for near-infrared detection measured (primarily) at RT. These CNT architectures with suspended CNTs [22–24] or vertically aligned CNTs [25] contained CNTs with multiple chiralities and numbers of walls with an averaged absorption effect. They have the potential for broadband absorption due to multiple absorption sites and can be integrated into array configurations for commercial applications. Our new designs were characterized at relatively low bias current, RT, and atmospheric pressure. It is noteworthy that the voltage responsivity of 30 V/W for sample 2 was already comparable to the responsivity of a vertically aligned MWCNT design obtained at very low temperature (84 K) and under vacuum conditions [25]. The responsivity of our miniaturized architecture in sample 2 was also competitive with larger bolometer architectures using suspended MWCNTs [23–24] and SWCNTs [22] (see Table 1). The new microbolometer had a fast response time of ≈0.15 ms for both configurations and was faster than the devices listed in Table 1. Sample 2 had a TCR of −0.91 %/K, which was much higher than that of sample 1 and, in most cases, comparable or higher than the state-of-the-art aligned bolometer architectures with pristine CNTs. Our samples showed reliable operation even at temperatures above 200 °C. In addition, the new design has the potential for the highest integration densities. Using a simple lithography process, minimized structures smaller than 5 × 5 μm^2^ can be achieved.

**Table 1:** Comparative analysis of microbolometer performance metrics. All values were obtained at room temperature (if not marked otherwise).

Microbolometer	|TCR|	ℜ*_V_* (V/W)	Response time (ms)

suspended SWCNTs film [22]	≈1 at 330 K	30 (at 100 mV)	50
suspended MWCNT film [24]	0.144	30 (at 300 μA )	4.4
suspended MWCNTs on polymer substrate [23]	0.08	40 (at 2 V )	800
suspended MWCNTs on Cr substrate [23]	0.08	110 (at 2.5 V)	60
vertical aligned MWCNT forest [25]	3.9 at 274 K	42 (at 1.5 mA and 84 K)	0.19
this work (sample 1)	0.22	13 (at 24 μA )	0.15
this work (sample 2)	0.91	30 (at 100 μA )	0.15

The effective temperature increase of our microbolometer with the highest responsivity (configuration 2) was Δ*T* = 2°C at *P*/*A* = 4 × 10^6^ W/m^2^ as shown in Figure 5b. Boldor [26] demonstrated for their multiwalled carbon nanotube layers the same local temperature increase of Δ*T* = 2 °C at a much smaller power density of *P*/*A* = 260 W/m^2^. Yaghoobi [27] demonstrated for their vertical CNT block a much higher temperature increase of Δ*T* = 1700 °C at *P*/*A* = 5 × 10^5^ W/m^2^, which is smaller than ours. Hence, several optimization steps are planned to improve the responsivity. The absorbed laser power was calculated very conservatively with an absorption area of *A* = 20 × 20 μm^2^. In reality, the bolometer resistance and responsivity are dominated by the large CNT wall resistance. By using and optimizing a lateral design with smaller absorption and stabilizing blocks, the lateral absorption area and the absorbed laser power can be reduced by more than one order of magnitude. Assuming a similar temperature increase in the CNT walls, the responsivity will increase by an order of magnitude. In addition, the CNT height can be reduced to the effective light absorption depth to achieve a more homogeneous temperature rise over the wall height, resulting in a larger resistance change and responsivity increase. CNT architectures with initially low thermal coupling at atmospheric pressure can reduce the thermal coupling to the environment in vacuum, leading to a significant improvement in bolometric photoresponse [24]. The first design of our bolometer used pristine CNTs. However, the high surface-to-volume ratio of CNTs is very advantageous for further coating with, for example, VO*_x_* or ZnO. Thus, this architecture provides a platform technology to increase the responsivity of the fabricated new 3D-based bolometer devices.

Additionally, the NEP value will be improved with increased ℜ*_V_*, allowing for the detection of weaker IR signals. These calculations indicate that the new CNT microbolometer could achieve superior performance in optimized micro-nano architectures with more efficient absorber region and under vacuum measurement conditions with higher bias current. Our cost-efficient new microbolometer can be suitable for fast characterization of small materials under optical as well as microwave and terahertz illumination.

## Conclusion

This work aims to advance the understanding and implementation of CNT-based non-cryogenic IR microbolometers, providing valuable insights for researchers, engineers, and industries seeking to harness the full potential of this new thermal sensing technology. A 3D micro-nano integrated CNT architecture has been utilized to develop a non-cryogenic IR microbolometer, enabling a minimum pitch dimension of less than 5 × 5 μm^2^ through a simple lithography process. This approach has significant potential for high-density integration. The device exhibited a rapid response time of ≈0.15 ms and a high negative TCR of −0.91 %/K. The new microbolometer operates reliably at temperatures even above 200 °C, demonstrating its robustness in extreme environments. We achieved a current (voltage) responsivity of 2 mA/W (30 V/W) to the absorbed IR power, which is already comparable in the context of current CNT-based microbolometer technology with high potential for miniaturized design and responsivity optimization. This study not only contributes to the current state of the art in microbolometer technology, but also opens avenues for further innovation and broader application in both scientific and consumer markets. Ongoing advances in CNT research and microbolometer technology hold the promise of efficient cost reduction as well as sensitive and versatile thermal detection devices suitable for a wide range of high-tech applications.

## Supporting Information

File 1 Additional experimental data.

## Data Availability

The data that supports the findings of this study is available from the corresponding author upon reasonable request.
