# Supplementary material for: Bolometric IR photoresponse based on a 3D micro-nano integrated CNT architecture
Source: Beilstein J Nanotechnol. 2024 Aug 15;15:1030–40. doi: 10.3762/bjnano.15.84 (PMC11331537; doi:10.3762/bjnano.15.84)
Supplement: File 1 — Additional experimental data. [file Beilstein_J_Nanotechnol-15-1030-s001.pdf]

BEILSTEIN JOURNAL OF NANOTECHNOLOGY

Supporting Information

for

Bolometric IR photoresponse based on a 3D micro-nano integrated CNT architecture

Yasameen Al-Mafrachi, Sandeep Yadav, Sascha Preu, Jörg J. Schneider and Oktay Yilmazoglu

Beilstein J. Nanotechnol. **2024,** *15,* 1030–1040. doi:10.3762/bjnano.15.84

Additional experimental data

License and Terms: This is a supporting information file under the terms of the Creative Commons Attribution License (https://creativecommons.org/licenses/by/4.0). Please note that the reuse, redistribution and reproduction in particular requires that the author(s) and source are credited and that individual graphics may be subject to special legal provisions.
The license is subject to the *Beilstein Journal of Nanotechnology* terms and conditions: (https://www.beilstein-journals.org/bjnano/terms)

SEM images of further devices

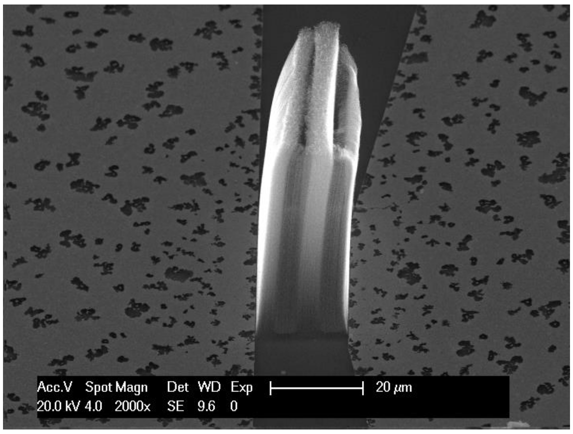

(a)

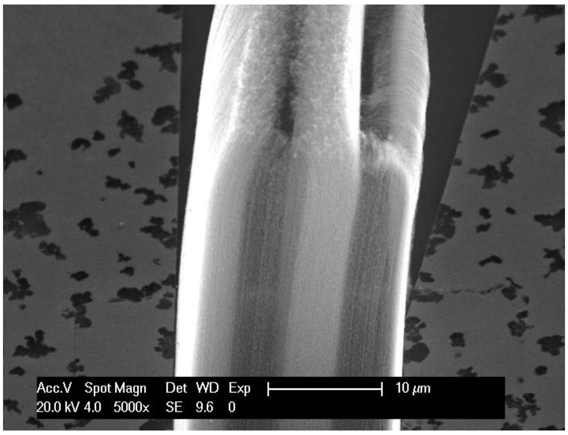

(b)

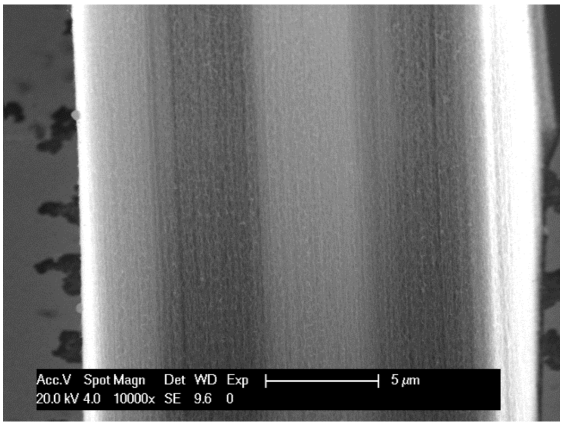

(c)

Figure S1: (a) SEM images of the additional device 3. (b,c) Device 3 with higher magnifications.

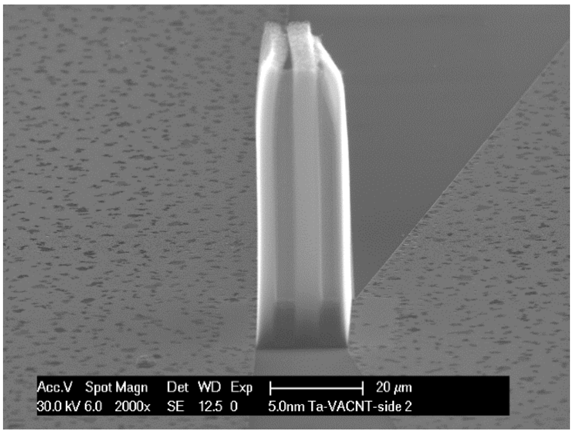

(a)

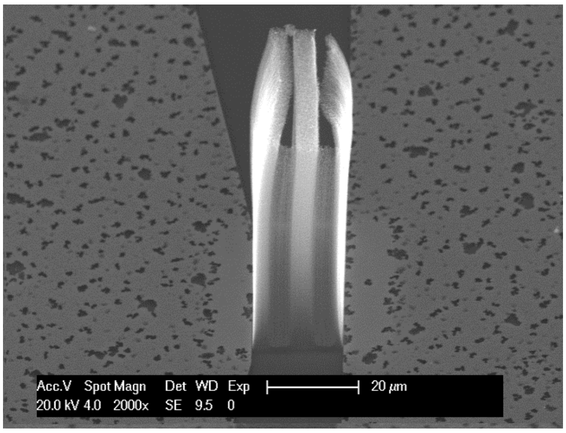

(b)

Figure S2: (a) SEM images of device 4 and (b) device 5.

Single oscilloscope measurements from device 3 using direct Au whisker contacts (configuration 2).

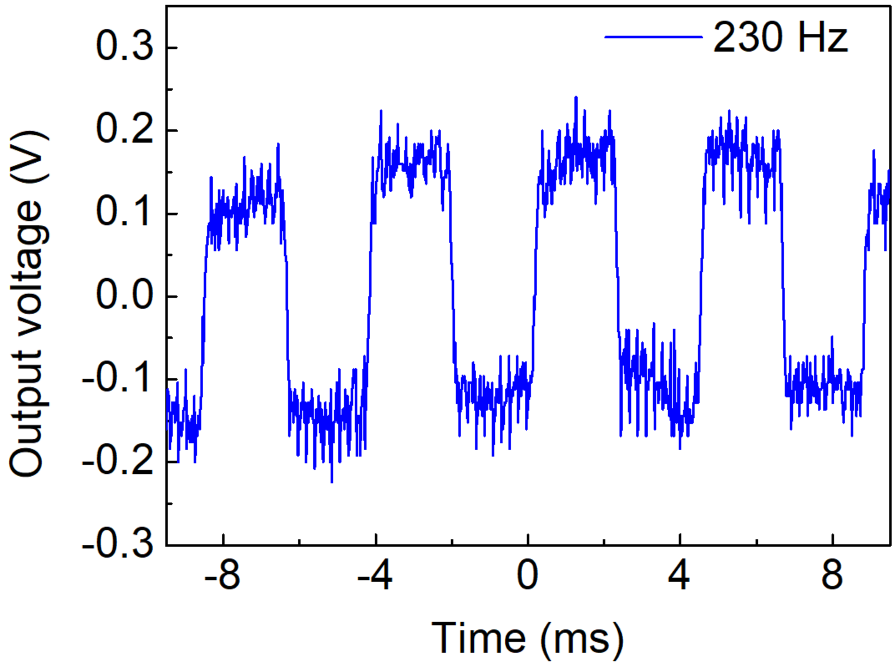

Figure S3 (a): Single response measurement of device 3 at 230 Hz chopping frequency.

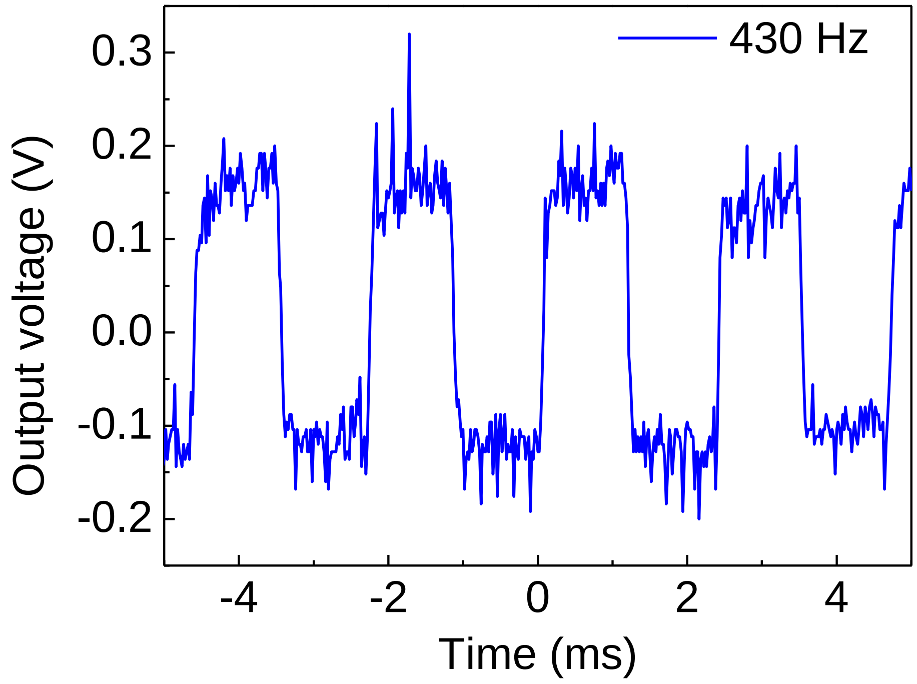

Figure S3 (b): Single response measurement of device 3 at 430 Hz chopping frequency.

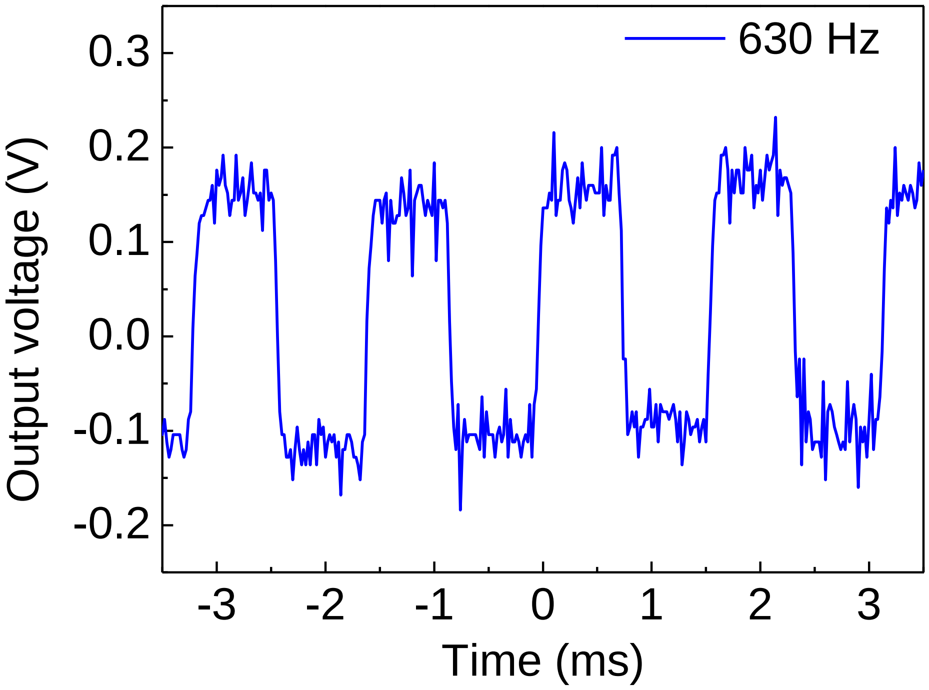

Figure S3 (c): Single response measurement of device 3 at 630 Hz chopping frequency.

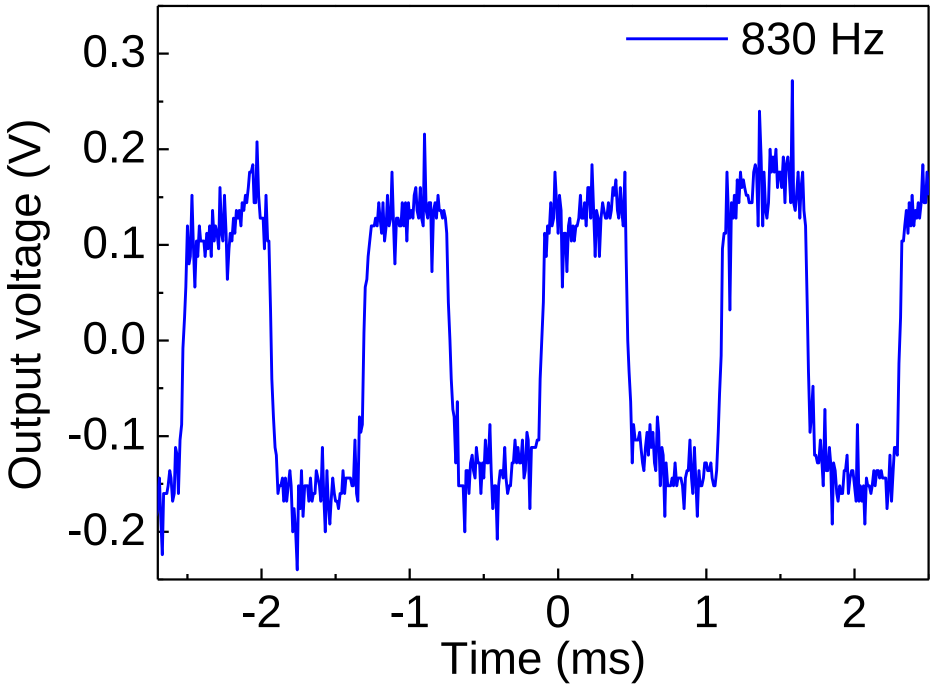

Figure S3 (d): Single response measurement of device 3 at 830 Hz chopping frequency.

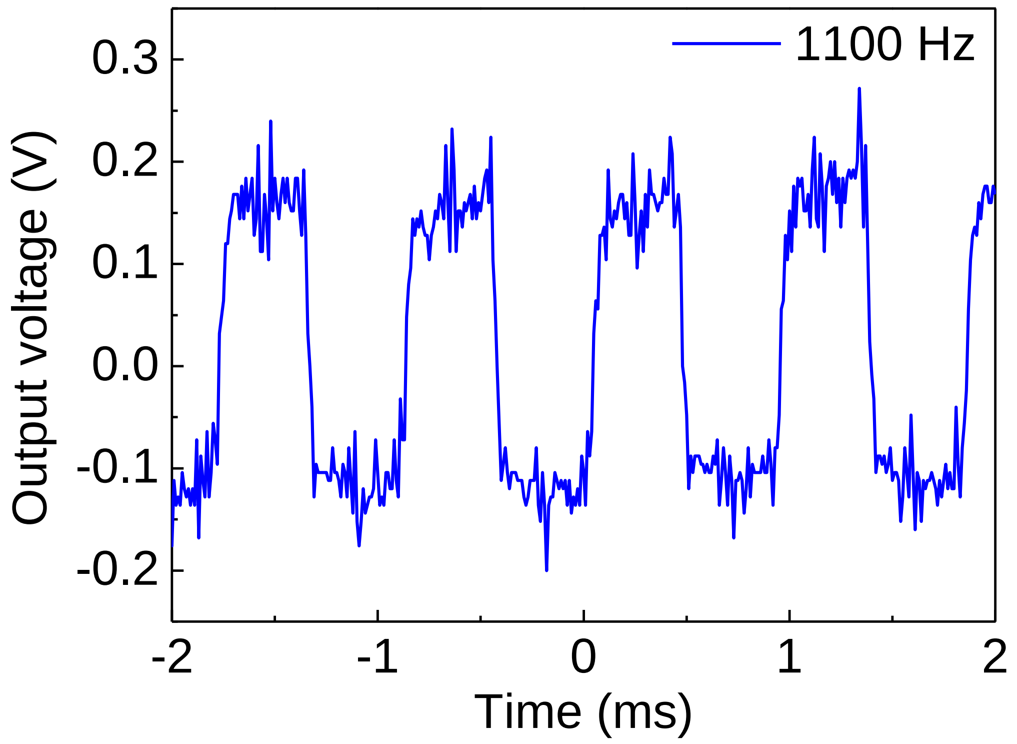

Figure S3 (e): Single response measurement of device 3 at 1100 Hz chopping frequency.

Single oscilloscope measurements from device 4 using direct Au whisker contacts (configuration 2).

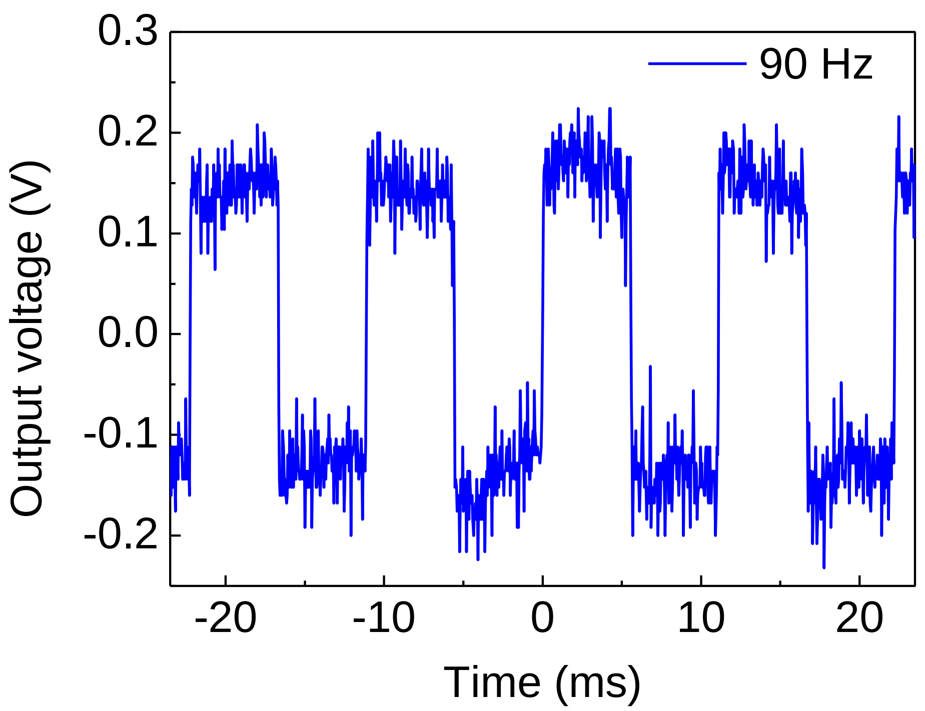

Figure S4 (a): Single response measurement of device 4 at 90 Hz chopping frequency.

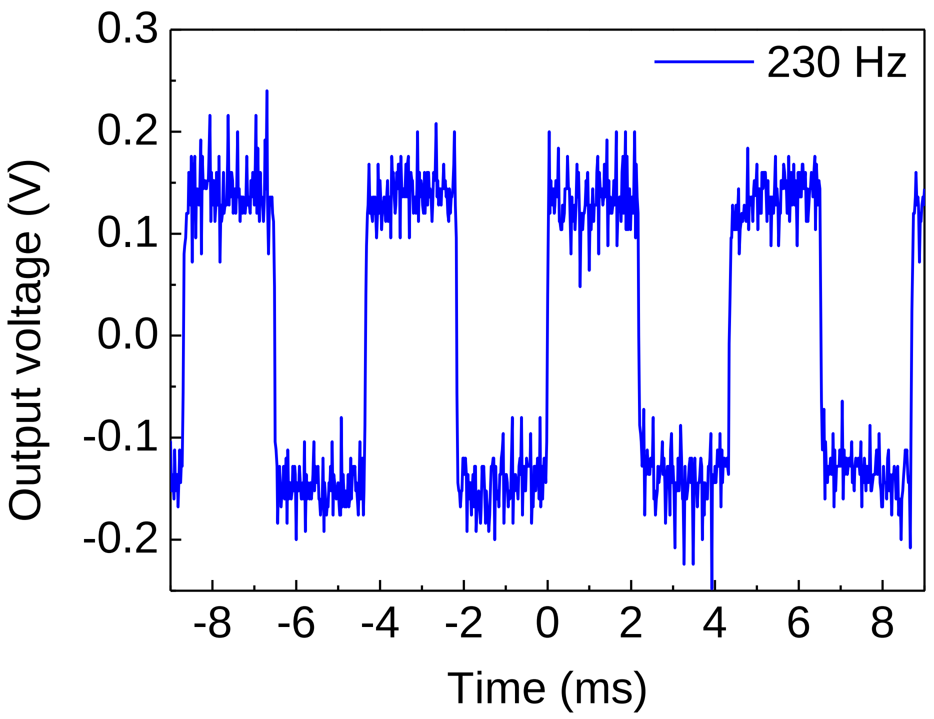

Figure S4 (b): Single response measurement of device 4 at 230 Hz chopping frequency.

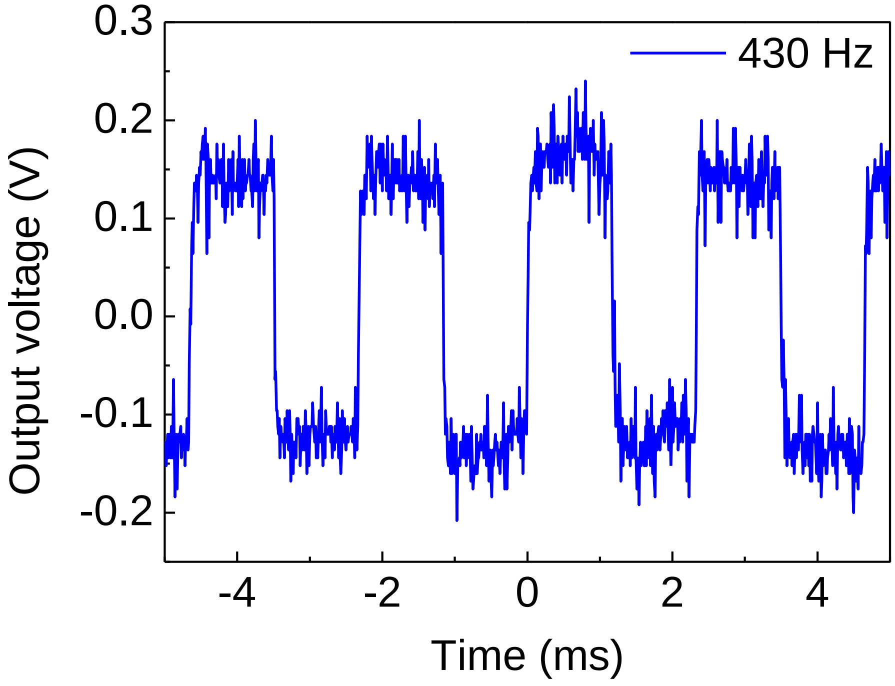

Figure S4 (c): Single response measurement of device 4 at 430 Hz chopping frequency.

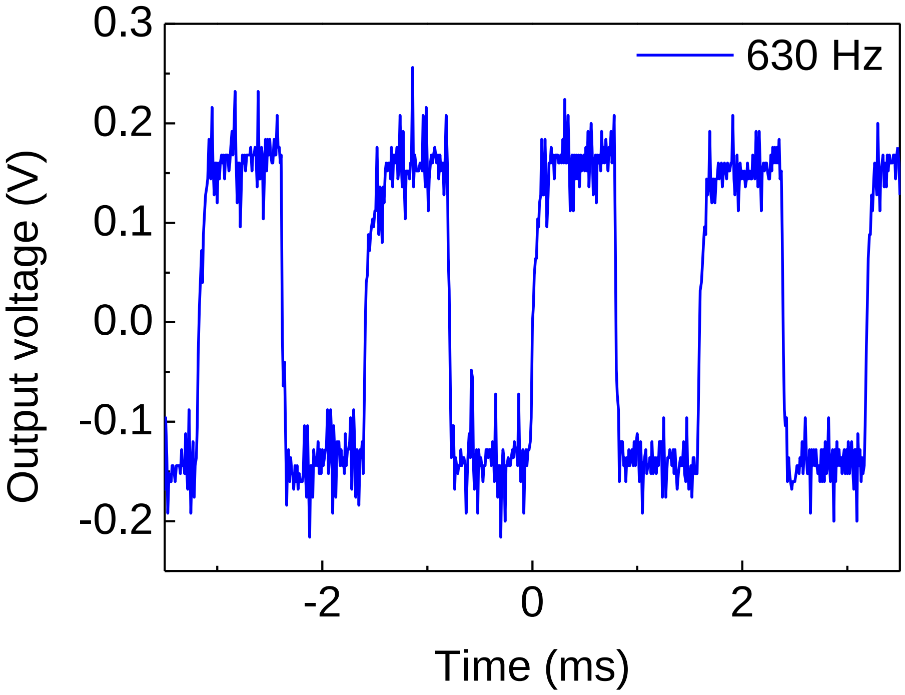

Figure S4 (d): Single response measurement of device 4 at 630 Hz chopping frequency.

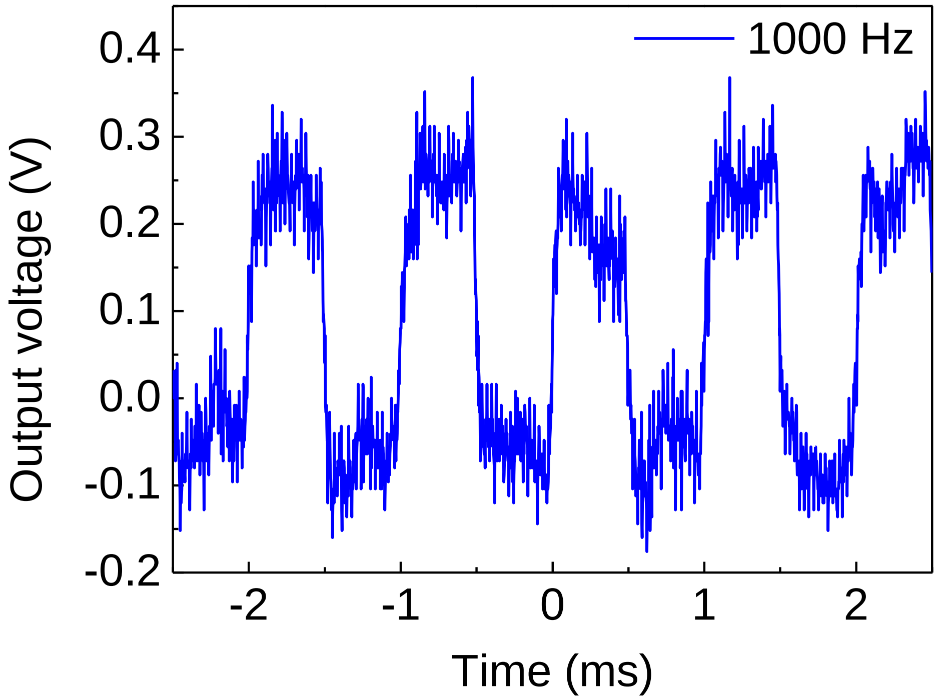

Figure S4 (e): Single response measurement of device 4 at 1000 Hz chopping frequency.

Single oscilloscope measurements from device 5 using direct Au whisker contacts (configuration 2).

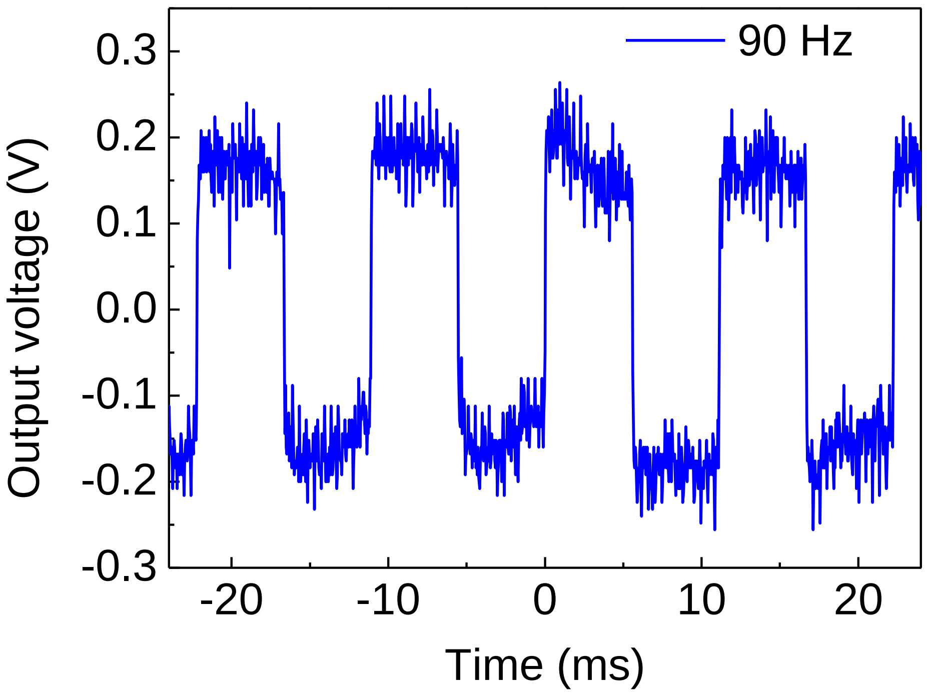

Figure S5 (a): Single response measurement of device 5 at 90 Hz chopping frequency.

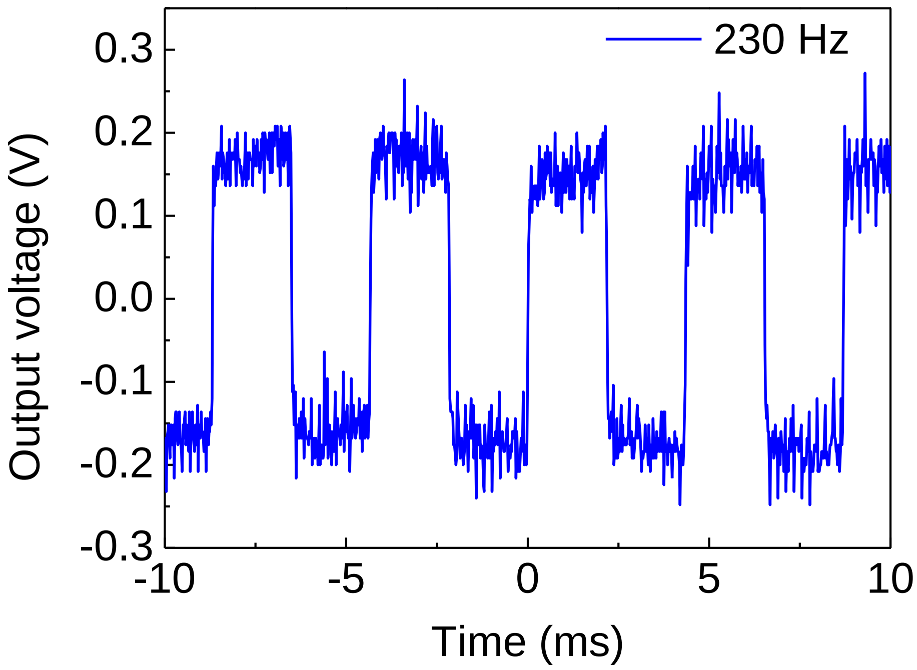

Figure S5 (b): Single response measurement of device 5 at 230 Hz chopping frequency.

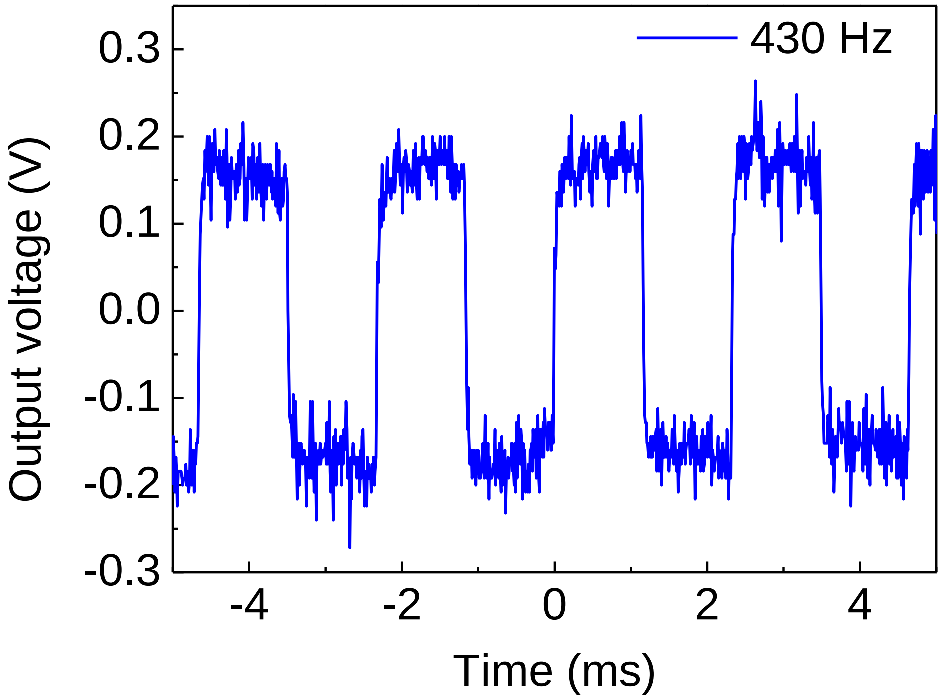

Figure S5 (c): Single response measurement of device 5 at 430 Hz chopping frequency.

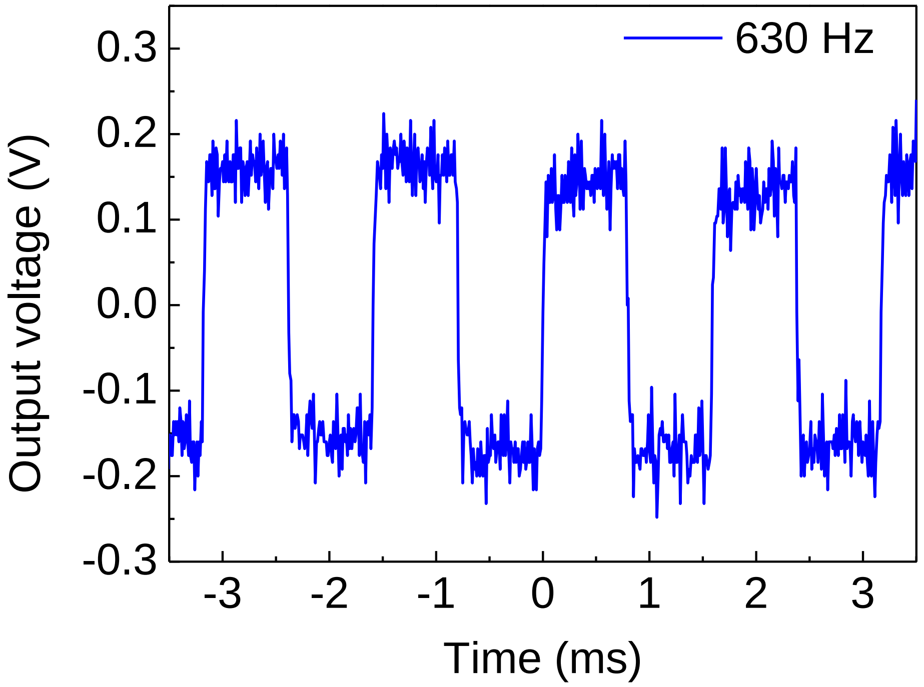

Figure S5 (d): Single response measurement of device 5 at 630 Hz chopping frequency.

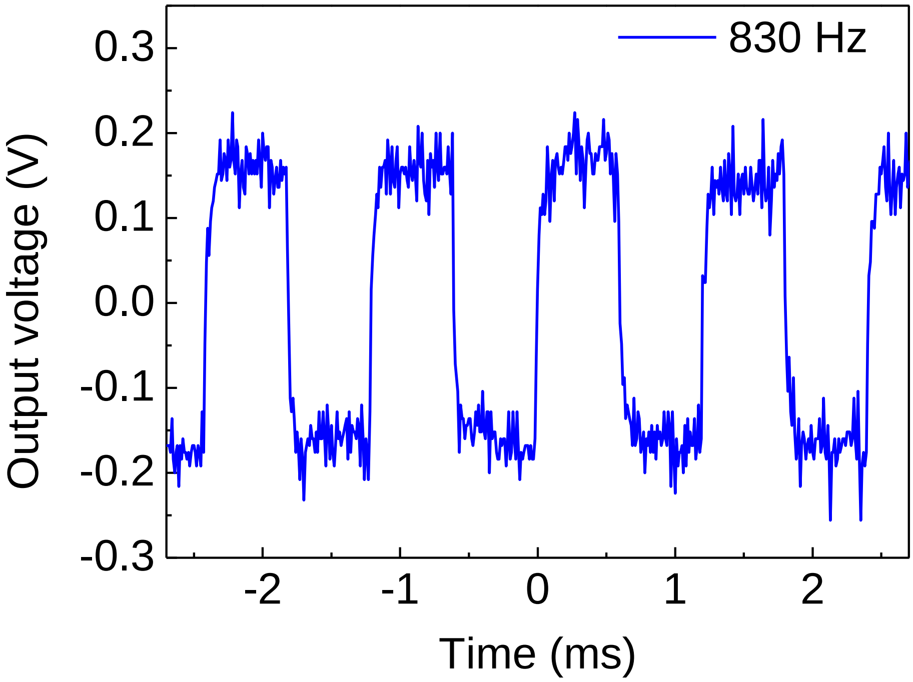

Figure S5 (e): Single response measurement of device 5 at 830 Hz chopping frequency.

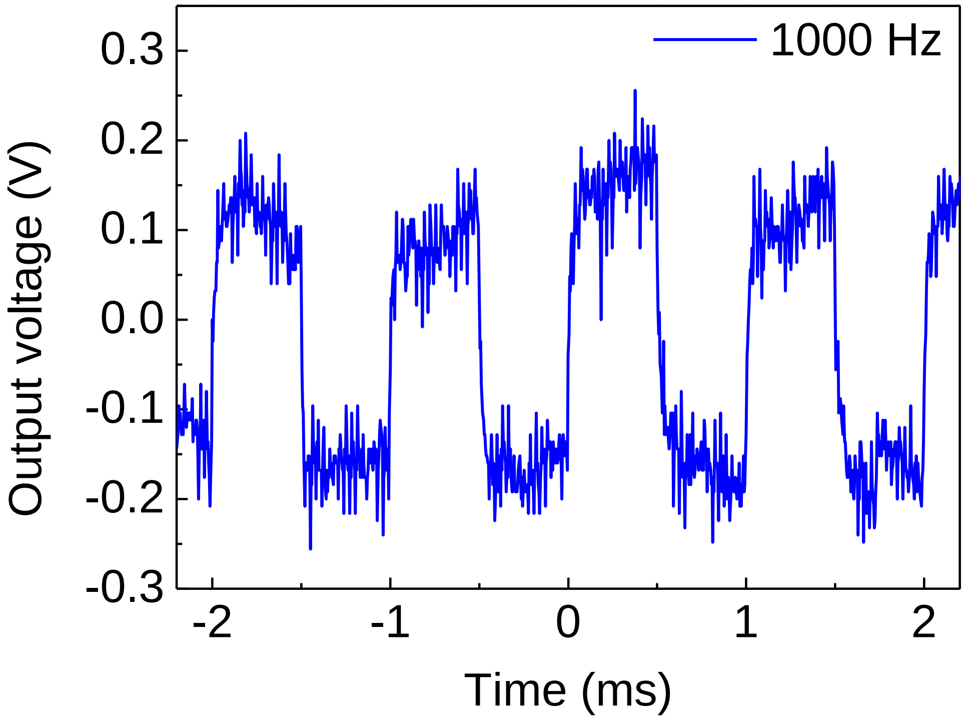

Figure S5 (f): Single response measurement of device 5 at 1000 Hz chopping frequency.
